# Commentary: Decomposition of Heart Rate Variability Spectrum into a Power-Law Function and a Residual Spectrum

**DOI:** 10.3389/fcvm.2018.00094

**Published:** 2018-07-26

**Authors:** Paolo Castiglioni

**Affiliations:** IRCCS Fondazione Don C. Gnocchi, Milan, Italy

**Keywords:** heart rate variability, 1/f slope, power spectral analysis, fractals, self-similarity

Recently J.Kuo and C.D.Kuo published a method for decomposing the power spectrum, *PSD*, of heart rate variability (HRV) in a power-law function “*displaying the fractal characteristics of the PSD*” and in a residual spectrum, rHRV (1). Readers of this journal should consider important methodological aspects of this approach to interpret correctly its results.

The method is based on plotting *PSD* vs. the frequency *Frq* in a log-log scale and on estimating the slope, *s*, and the intercept, *Y*, of the regression line, *rg*, fitting log(*PSD*) vs. log(*Frq*):

(1) $$\begin{array}{r} rg=s\times log\left(Frq\right)+Y \end{array}$$

Then the regression line is anti-transformed to obtain the power law component, *PSD*_*rg*_:

(2) $$\begin{array}{r} PSD_{rg}=10^{s\times \log\left(Frq\right)+Y} \end{array}$$

and the residual, *rPSD*, defined as:

(3) $$\begin{array}{r} rPSD=\frac{PSD}{PSD_{rg}} \end{array}$$

The authors express *rPSD* in units of the power spectrum (ms^2^/Hz if the tachogram is measured in ms), assuming *PSD*_*rg*_ to be a dimensionless quantity. However, any regression line has the units of the data that it fits. Therefore, *rg* in Equation (1) has the units of log(*PSD*) and after the anti-transformation, *PSD*_*rg*_ has the units of 10^log(*PSD*)^, i.e., of *PSD*. This means that *PSD*_*rg*_ is expressed in ms^2^/Hz and that the spectral ratio *rPSD* in Equation (3) is a dimensionless number

This explains why *PSD*_*rg*_ shows the same range of values of the original spectrum in Figure 1C of (1). This also explains the surprisingly very low values of *rPSD* in Figure 1D and of the *rHRV* high frequency (HF) power in Figure 2. In fact, very low values at the higher frequencies are surprising if obtained after removal from the original spectrum of a power-law component whose contribution should be minimal at the higher frequencies. But actually, the statistical comparison between powers of *HRV* and powers of *rHRV* in the left panels of Figure 2 has no meaning, because the two quantities have different units. The authors suggest in their conclusions that “*the clinical meaning and significance of rHRV measures might be different from traditional HRV measures*”: this is certainly true because the *rHRV* measures introduced in (1) cannot be considered measures of HRV power.

A second methodological aspect deserves to be commented on. The authors fit the regression line up to the Nyquist frequency, i.e., the highest frequency of the spectrum. Starting from the first report of a “1/f” trend in the HRV spectrum (2), the literature considered only frequencies lower than the low frequency (LF) band to exclude non-fractal components, like the respiratory sinus arrhythmia and the Mayer waves, from the regression fitting (3). For this reason, *PSD*_*rg*_ cannot be considered the true “fractal component” of the spectrum, because the regression slope *s* is influenced by oscillations in the LF and HF bands. International guidelines on HRV recommended estimating the “1/f” trend only on frequencies <0.04 Hz (4). This requires long-term recordings and the short duration of the HR series considered in this work (512 beats, i.e., less than 7 min) does not allow estimating the regression slope on a sufficient number of spectral lines. It should be considered, however, that an alternative method allows extracting power-law fractal components even from HRV spectra estimated on similarly short data segments. This is possible by exploiting the fact that coarse graining the tachogram preserves the self-similar dynamics of HR and not the LF and HF periodic oscillations (5). The coarse-graining method is used not only to quantify the fractal component of HR even from relatively short segments of data (6), but also to better estimate the LF and HF oscillatory components by removing the underling fractal power (7, 8).

In conclusion, the *rPSD* function proposed in (1) appears an interesting way to quantify deviations from the power-law trend. However, it cannot be considered a measure of spectral power, and it may be still affected by HR fractal components if the least-square fitting includes the LF band.

## Author contributions

The author confirms being the sole contributor of this work and approved it for publication.

### Conflict of interest statement

The author declares that the research was conducted in the absence of any commercial or financial relationships that could be construed as a potential conflict of interest.
